# Hepatitis C outreach project and cross-sectional epidemiology in high-risk populations in Trondheim, Norway

**DOI:** 10.1177/20499361211053929

**Published:** 2021-10-28

**Authors:** Raisa Hannula, Jonas Söderholm, Therese Svendsen, Maja Skaland, Svein A. Nordbø, Harald Steinum, Jan K. Damås

**Affiliations:** Department of Infectious Diseases, Trondheim University Hospital, 7006 Trondheim, Norway; Division of Clinical Microbiology, Department of Laboratory Medicine, Karolinska Institutet, Karolinska University Hospital Huddinge, Stockholm, Sweden; Department of Infectious Diseases, Trondheim University Hospital, Trondheim, Norway; Department of Infectious Diseases, Trondheim University Hospital, Trondheim, Norway; Department of Medical Microbiology, St. Olavs Hospital HF, Trondheim University Hospital, Trondheim, Norway; Department of Clinical and Molecular Medicine, Norwegian University of Science and Technology, Trondheim, Norway; Department of Infectious Diseases, Trondheim University Hospital, Trondheim, Norway; Department of Infectious Diseases, Trondheim University Hospital, Trondheim, Norway

**Keywords:** community, epidemiology, hepatitis C virus, high-risk populations, immigrants, outreach, people who use drugs, prevalence, prisoners

## Abstract

**Background:**

Hepatitis C is highly prevalent among people who use drugs (PWUD), and the hepatitis C virus (HCV) epidemic is less characterised in Norway. The aims of the study were to assess the prevalence and treatment willingness in high-risk populations by reaching out to frequently visited sites for high-risk populations.

**Methods:**

Individuals from high-risk populations were included from September 2015 to March 2017. Two dedicated study nurses frequently visited the local opioid substitution clinic, outpatient clinics, PWUD day centres, local prison, and refugee centre in Trondheim, Norway. Demographic data, risk behaviour, and clinical symptoms were obtained by study questionnaire. Subjects with anti-HCV^+^ rapid test were subsequently tested for HCV RNA and genotyped. Viraemic patients were offered referral for HCV treatment evaluation.

**Results:**

A total of 381 participants were included in the study: 52 immigrants, 62 prisoners, and 267 PWUD. The anti-HCV prevalence rates were 0% (*n* = 0) in immigrants, 40% (*n* = 25) in prisoners, and 61% (*n* = 164) in PWUD, with 24% (*n* = 15) of prisoners and 42% (*n* = 108) of PWUD being viraemic. Of those qualifying for treatment (*n* = 31), 30 wished to be evaluated.

**Conclusion:**

This study showed high HCV prevalence in prisoners and PWUD and that infected high-risk patients were interested in treatment evaluation.

## Introduction

The hepatitis C virus (HCV) infects the liver and is transmitted through contaminated blood. Approximately 69 million people worldwide are estimated to have chronic HCV infection.^1^ Today, HCV is mainly transmitted among people who use drugs (PWUD),^2^ and the anti-HCV antibody prevalence in this group is estimated to be greater than 60%.^3^ Two approaches to limit the spread of new infections in this high-risk population are needle exchange programmes (NEPs) and oral opiate substitution therapy (OST).^4^ HCV treatment during the interferon era achieved virologic cure rates between 40% and 80%, with treatment durations for ⩾24 weeks depending on the genotype and stage of the disease and were associated with adverse events.^5^ Today, patients can be treated using interferon-free, all-oral, pan-genotypic direct-acting antivirals (DAAs) that can cure most patients with only 8–12 weeks of therapy with adverse event profiles similar to placebo.^6^ Real-world evidence has shown similar high sustained virologic response (SVR) rates for patients either in OST or NEPs as for patients in the clinical trials.^7^ However, in order to initiate antiviral treatment, the healthcare system needs to reach the patients. One obstacle for patients voluntarily reaching out to specialist departments for antiviral therapy is low awareness of HCV status among PWUD.^8^ In addition, their general trust in the healthcare system is low, and PWUD do not necessarily see HCV as an urgent medical concern.^9^

The prevalence of HCV antibody positivity in Norway is estimated to be between 0.5% and 0.7% in the capital region of Oslo,^10^ with two studies (from 2000 and 2009, respectively) showing 0.2% HCV prevalence in northern Norway to 0.7% in 11 Norwegian counties.^11,12^ In contrast, studies from other countries have reported the prevalence of HCV antibody positivity to be 1.9% in immigrants,^13^ 7.4% in prisoners,^14^ and 33% in PWUD,^15^ thus the local HCV treatment guidelines at the time suggested these groups to be high-risk populations.^16^ However, updated Norwegian data on HCV prevalence in high-risk populations are scarce. A recent study suggested that a multifaceted approach with screening, prevention, and treatment is necessary to reach the World Health Organization (WHO) elimination goal.^1^ A national Norwegian hepatitis C action plan was published in 2018.^17^ It is essential to have reliable epidemiological data from different risk groups in several regions to make correct choices in implementing this plan. The aims of this study were to use an approach where high-risk populations were reached by offering mobile HCV screening, as well as to evaluate the HCV prevalence and epidemiology in high-risk populations (immigrants, prisoners, and active or former PWUD) in Trondheim, Norway.

## Materials and methods

### Patients, enrolment, and outreach

The study population consisted of three different high-risk groups.^16^ In the first group, immigrants were enrolled in adjunction to voluntary information meetings at the local Trondheim refugee centre. Different language groups were invited, through readily available posters and leaflets in relevant languages for people present at the centre, to separate meetings with an interpreter in attendance. In the second group, incarcerated individuals were informed at the local prison in Trondheim. Study information was accessible on a poster, and leaflets were handed out. The prison healthcare department also provided information. In the third group, PWUD with either previous or current risk behaviour for HCV exposure were actively pursued at the OST clinic, PWUD housing, the Trondheim NEP, outpatient clinics, and PWUD day centres. Enrolment in the study was on voluntary basis without compensation. Informed consent forms in Norwegian, Arabic, English, Somali, or Tigrinya were available. Written informed consent was obtained prior to enrolment, and informed consent could not be signed by individuals noticeably influenced by drugs. The study aimed to enrol 400 patients, which was one quarter of the estimated at-risk population in Trondheim. The study was approved by the Regional Ethical Review Board, REK Central, Trondheim, Norway (2015/473/REK midt).

Two dedicated study nurses from the infectious disease department at the Trondheim University Hospital frequently visited places in the community and the hospital outpatient clinic for inclusion. Based on the averages during the months preceding the study period, Trondheim municipality estimated the high-risk populations to consist of 1,000 PWUD, 300 receiving OST, 200 prisoners, and 300 immigrants. Since individuals can move between groups the total estimated number was reduced to 1,600. The aim was to include 1/4 of the total at-risk population (*n* = 400). Participants were included from September 1, 2015, to March 15, 2017. After signing an informed consent form, demographic data, risk behaviour, and clinical symptoms were obtained using the in-house developed study questionnaire (Table 1) that was approved by the ethical committee before use. Liver elastography assessment was offered onsite during the second study visit to respective enrolment site to HCV RNA^+^ patients using a mobile FibroScan^®^ 402 (Echosens, Paris, France). Initially, only a medium probe was available, but an extra-large probe was obtained and used starting September 6, 2016. The degree of fibrosis was defined in the study as no/minimal fibrosis (F0–F1 < 7 kPa), moderate/advanced fibrosis (F2–F3 = 7–12.4 kPa), and cirrhosis (F4 ⩾ 12.5 kPa).^18^

**Table 1.** Study questionnaire – survey cohorts.

*n* (%)	Immigrants (*n* = 52)	Prisoners (*n* = 62)	PWUD (*n* = 267)	Total
History of IDU				
Yes	0 (0)	32 (52)	225 (84)	257 (67)
No	24 (46)	30 (48)	40 (15)	94 (25)
No answer	28 (54)	0 (–)	2 (1)	30 (8)
Duration of IDU^a^				
<10 years	N/A	12 (23)	83 (37)	95 (25)
⩾10 years	N/A	19 (59)	130 (58)	149 (39)
No answer	N/A	1 (3)	12 (5)	13 (3)
Sharing needles during the last 4 weeks^a^				
Yes		2 (3)	23 (10)	25 (7)
No		30 (48)	189 (84)	219 (57)
Unknown		0 (–)	13 (6)	13 (3)
Sharing equipment during the last 4 weeks^a^				
Yes		1 (3)	29 (13)	30 (8)
No		31 (97)	188 (84)	219 (57)
Unknown		0 (–)	8 (4)	8 (2)
Current OST				
Yes	0 (0)	8 (13)	84 (31)	92 (24)
No	23 (44)	53 (85)	182 (68)	258 (68)
No answer	29 (56)	1 (2)	1 (<1)	31 (8)
Previous HCV treatment	N/A			
Yes		6 (3)	28 (10)	34 (9)
No		54 (87)	237 (89)	291 (76)
No answer		2 (3)	2 (1)	4 (1)
Previous HCV treatment outcome^b^				
Achieved SVR		0 (–)	19 (68)	19 (5)
Relapse or reinfection		0 (–)	2 (7)	2 (1)
Discontinued		3 (50)	5 (18)	8 (2)
Unknown		3 (50)	1 (4)	4 (1)
No answer		0 (–)	1 (4)	1 (<1)
Aware of HCV transmission				
Yes	N/A	20 (33)	134 (50)	154 (40)
No	N/A	6 (10)	24 (9)	30 (8)
No answer	N/A	36 (58)	109 (41)	145 (36)
MODE of transmission among patients with a history of IDU				
IDU	N/A	23 (72)	115 (51)	138 (36)
Sexual	N/A	0 (–)	4 (2)	4 (1)
Tattoo/piercing	N/A	0 (–)	1 (<1)	1 (<1)
HCP accident	N/A	0 (–)	1 (<1)	1 (<1)
Unknown	N/A	2 (6)	0 (–)	2 (1)
No answer	N/A	7 (22)	74 (33)	81 (21)
HIV test during the last 12 months				
Yes	7 (13)	11 (18)	91 (34)	109 (29)
No	10 (19)	39 (63)	138 (52)	187 (49)
Unknown	35 (67)	11 (18)	35 (13)	81 (21)
No answer	0 (–)	1 (2)	3 (1)	4 (1)
HCV test during the last 12 months				
Yes	3 (6)	16 (26)	111 (42)	130 (34)
No	8 (15)	37 (60)	118 (44)	163 (43)
Unknown	40 (77)	8 (13)	37 (14)	85 (22)
No answer	1 (2)	1 (2)	1 (<1)	3 (1)

HCP, Healthcare professional; HCV, hepatitis C virus; HIV, human immunodeficiency virus; IDU, injecting drug user; NA, not applicable; OST, opiate substitution therapy; PWUD, people who use drugs; SVR, sustained virologic response.

a With history of IDU.

b With previous HCV treatment.

### Laboratory analyses

Individuals without a recent confirmed positive anti-HCV test were tested for HCV antibodies using the OraQuick^®^ HCV rapid antibody test (OraSure Technologies Inc., Bethlehem, PA, USA).^19^ All positive samples were retested using standard HCV serology (anti-HCV, Abbott Architect, Abbott Park, IL, USA) at the Trondheim University Hospital. All samples testing positive for anti-HCV were extracted for RNA (NucliSENS easyMAG, bioMerieux, and France), analysed for HCV RNA (Cobas^®^ 6800, Roche Diagnostics, Mannheim, Germany), and genotyped at the Department of Medical Microbiology, Trondheim University Hospital by Sanger sequencing according to the method described by Tong *et al.*^20^ Serology with respect to hepatitis B virus and HIV were analysed locally in addition to general clinical chemistry tests.

## Results

### Outreach project feasibility

Immigrants received information about hepatitis C from the study nurses in several voluntary meetings for different language groups over the study inclusion period. An interpreter for Arabic, Somali, or Tigrinya attended the meetings, and the consent forms were available in these languages in addition to Norwegian and English. All immigrants who wished to enrol in the study were tested at the same time at the refugee centre. The aim was to test 300 immigrants, which was based on the average number provided by the centre for the time before the study. All immigrants who attended the meetings signed up to the study. The large numbers of immigrants in 2015 declined in the months before the start of the study and only 52 immigrants were enrolled.

Prisoners allowed to move freely inside the prison premises were easier to reach than prisoners not allowed to move freely because they could easily be gathered to receive study information. The prisoners who were not allowed to attend meetings were informed by the posters and by the prison personnel. The recruitment of PWUD was more cumbersome; there was a high initial interest that was followed by failure to keep appointments. Thus, in order to gain data, the liver elastography assessments were in many cases done in adjunction to the initial study visit, despite this being in deviation with the study protocol. Only elastography results from viraemic patients were recorded in the case report form.

### Enrolled individuals – survey cohorts

The study enrolled 381 individuals, of which 52 were immigrants, 62 were prisoners, and 267 were PWUD, which was just below the target 400 individuals. Most individuals in each group were males (immigrants, 65%; prisoners, 85%; PWUD, 69%), with the lowest median age in immigrants (immigrants, 29 years (range, 18−61); prisoners, 33 years (range, 20−58); PWUD, 46 years (range, 19−72)). Immigrants were mainly from Eritrea (58%), followed by Sudan (15%), Somalia (15%), and Iraq (6%) (Supplemental Table 1).

No immigrant reported a history of injection drug use (IDU), compared with 52% (*n* = 32) of prisoners and 84% (*n* = 225) of PWUD. Almost all prisoners and PWUD who reported IDU answered that they had not shared any needles (prisoners, *n* = 30; PWUD, *n* = 189) or other equipment (prisoners, *n* = 31; PWUD, *n* = 188) during the last 4 weeks. Around half of the prisoners and PWUD reported IDU ⩾ 10 years. Thirteen percent (*n* = 8) of prisoners and 31% (*n* = 84) of PWUD reported currently participating in OST. At the time of the study, 20 (33%) of the prisoners and 134 (50%) of the PWUD were aware of HCV transmission per the study questionnaire, but many did not respond to the question (Table 1). For patients with a history of IDU, IDU was the mostly reported route of HCV transmission for both prisoners and PWUD (Table 1). Six (3%) prisoners and 28 (10%) PWUD recalled previous HCV treatment, with no prisoners and 19 PWUD reported having previously achieved SVR. Six of these were now viraemic and in total had 12 of the 34 previously treated detectable HCV RNA. Most responders reported not being tested for HIV during the last 12 months, but 111 (42%) of PWUD had an HCV test during the last 12 months (Table 1).

### Anti-HCV-positive individuals

**Figure 1.**
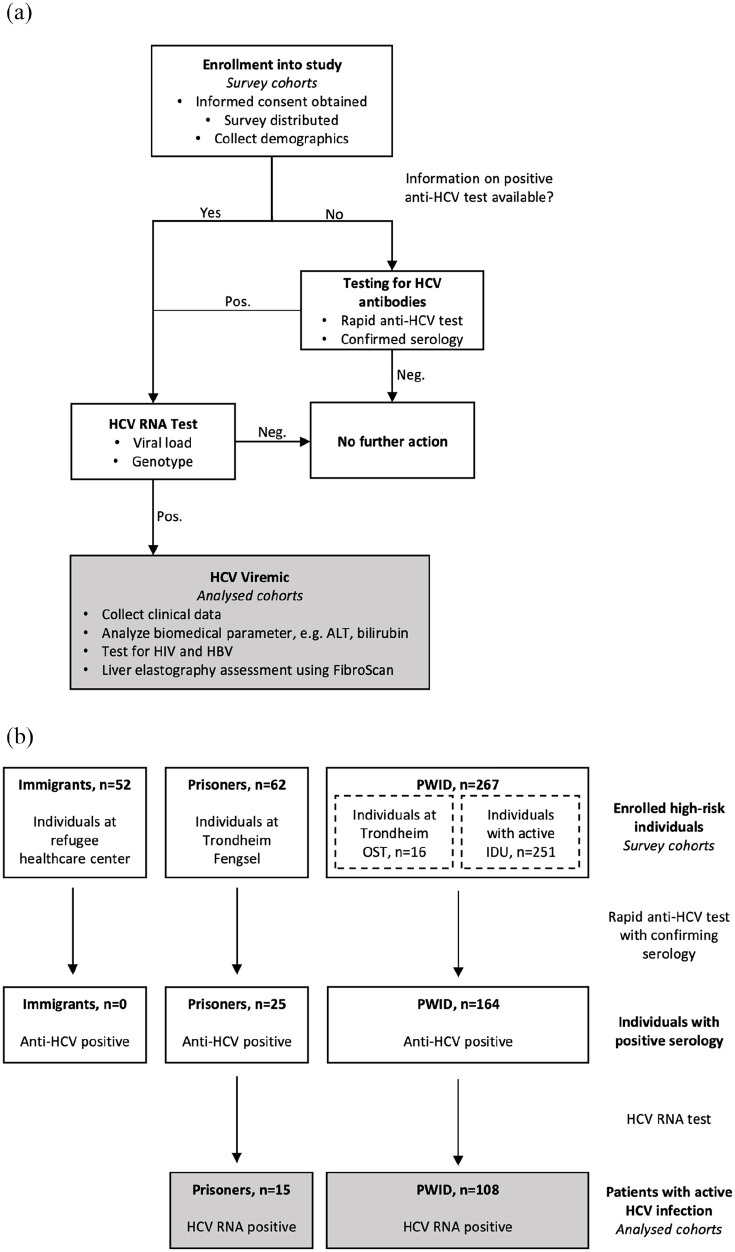
Study design: (a) study procedure and (b) flow chart for the study. Grey boxes are the analysed cohort. ALT, alanine aminotransferase; HBV, hepatitis B virus; HCV, hepatitis C virus; PWUD, people who use drugs.

**Figure 2.**
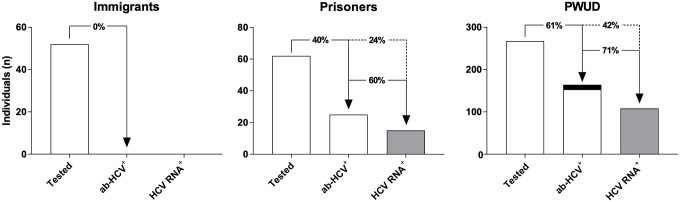
HCV prevalence anti-HCV and HCV RNA prevalence in immigrants, prisoners, and PWUD. Solid line prevalence per analysis and dashed line prevalence in previous analysis. Grey bars are analysed cohorts. Black bar indicates ab-HCV^+^ individuals with failed HCV RNA-test. HCV, hepatitis C virus.

No immigrant had a positive rapid anti-HCV test, so this group was not further analysed. Twenty-five prisoners and 163 PWUD tested positive in the rapid anti-HCV test. One PWUD had a recent anti-HCV^+^ test and was determined to be anti-HCV^+^ without requiring a rapid anti-HCV test, totalling 164 anti-HCV^+^ PWUD. All positive rapid anti-HCV tests were confirmed by serology (100% concordance). Thus, the anti-HCV prevalence was 40% (*n* = 25) among prisoners and 61% (*n* = 164) among PWUD (Figures 1 and 2). Of the HCV-positive patients, 76% (*n* = 19) of the prisoners and 69% (*n* = 113) of the PWUD had a previously confirmed anti-HCV test. Of those 34 who reported previous HCV treatment were 12 (35%) viraemic, indicating either re-infection or treatment failure. All previous treatment with available documentation at our hospital was interferon based.

### HCV viraemic patients – analysed cohorts

**Figure 3.**
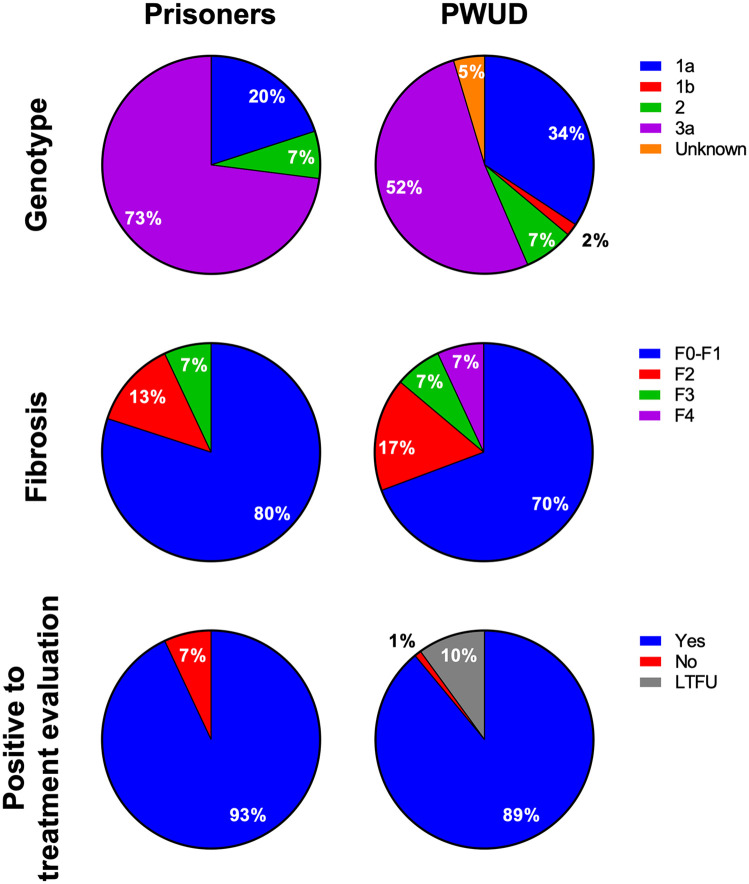
Patient assessments genotypes in prisoners (*n* = 15) and PWUD (*n* = 108). Fibrosis assessed by liver elastography in prisoners (*n* = 15) and PWUD (*n* = 89). Prisoners (*n* = 15) and PWUD (*n* = 108) positive to treatment evaluation. LTFU, lost to follow-up; PWUD, people who use drugs.

Owing to sampling difficulties and noncompliance, 11 PWUD could not be analysed for HCV RNA, leaving 153 PWUD to be analysed for HCV RNA. Of the tested anti-HCV^+^ individuals, 15 (60%) prisoners, and 108 (71%) PWUD were HCV RNA^+^, resulting in HCV viraemic prevalence of 24% in prisoners and 42% in PWUD (Figure 2). Most HCV RNA^+^ patients were males (prisoners, 80%; PWUD, 72%) aged around 40 years (prisoners, 38 years (range, 21−57)); PWUD, 44 years (range, 19−72)). All patients with HCV, except two prisoners and six PWUD, were born in Norway. The median body mass index (BMI) was 27.4 kg/m^2^ (range, 22.1−34.0) for prisoners and 25.0 kg/m^2^ (range, 17.6−48.6) for PWUD. The most common genotype was 3a in both prisoners (73%) and PWUD (52%), with genotype 1a being the second most common in both cohorts (Figure 3). All but one of the patients who were eligible for treatment wanted a referral for further evaluation.

All prisoners and 89 HCV RNA^+^ PWUD had liver elastography performed. The median liver elasticity was 5.1 kPa (range, 3.0−11.2) in prisoners and 6.0 kPa (range, 1.5−34.3) in PWUD, reflecting that most patients in both groups had minimal or no fibrosis (F0–F1) (Figure 3). Liver cirrhosis (F4) was detected in 7% (*n* = 6) of PWUD and in no prisoners. At the time of the study in Norway, treatment was only offered to patients with moderate fibrosis and above (F2–F4). Of the 31 patients qualifying for HCV treatment, all but one wished to be evaluated for treatment at the hospital. The F2–F4 restriction for treatment was lifted in Norway in 2018, and for all the cohorts, 93% of all HCV RNA^+^ prisoners and 89% of HCV RNA^+^ PWUD wanted HCV treatment (Figure 3). No patient was HIV^+^, but 20% (*n* = 3) of the HCV RNA^+^ prisoners and 44% (*n* = 47) of the HCV RNA^+^ PWUD were anti-hepatitis B core (HBc) positive. However, no prisoners and only 1% (*n* = 1) of the PWUD were hepatitis B surface antigen (HBsAg) positive (Table 2).

**Table 2.** Demographics and test results in the analysed cohort.

	Prisoners (*n* = 15)	PWUD (*n* = 108)
Sex (males)	12 (80)	78 (72)
Age (median, range)	38 (21–57)	44 (19–72)
Born in Norway	13 (87)	102 (94)
BMI (range) [kg/m^2^]	27.4 (22.1–34.0)	25.0 (17.6–48.7)
Viral load, median (range) (IU/mL)	419,000 (105,000–6,970,000)	1,055,000 (40–34,500,000)
Liver elastography, median (range) (kPa)	5.1 (3.0–11.2)	6.0 (1.5–34.3)
HIV		
Positive	0 (–)	0 (–)
Negative	15 (100)	106 (98)
Not tested	0 (–)	2 (2)
Anti-HBc		
Positive	3 (20)	47 (44)
Negative	12 (80)	56 (52)
Not tested	0 (–)	5 (5)
HBsAg		
Positive	0 (–)	1 (1)
Negative	15 (100)	104 (96)
Not tested	0 (–)	3 (3)
Anti-HBs		
Positive	6 (40)	50 (49)
Negative	9 (60)	53 (49)
Not tested	0 (–)	5 (5)

BMI, body mass index; HBc, hepatitis B core; HBs, hepatitis B surface; HBsAg, hepatitis B surface antigen; HIV, human immunodeficiency virus; IU, international unit; kPa, kilo Pascal; PWUD, people who use drugs.

## Discussion

The DAA revolution has rapidly transformed treatment of HCV to a point where HCV elimination, according to the WHO’s definition, is an achievable goal in several countries.^21^ However, a barrier to treatment of high-risk populations is limited healthcare contact.^22^ The outreach model in this study showed that HCV evaluation and treatment initiation of PWUD might be feasible if specialist clinics cooperate with community nurses by offering appointments with infectious disease nurses in the community. This may provide treatment to patients who would not comply with treatment in a specialist outpatient clinic.

Because PWUD frequently serve as a reservoir for infecting others through needle sharing,^23^ treatment efforts in this cohort are essential to reaching the WHO elimination goal by 2030. Interestingly, the study showed a great interest in HCV treatment, with all but one treatment-eligible patients wanting further treatment evaluation, in contrast to other studies in PWUD.^9^ It is possible that awareness of interferon-free treatment alternatives has improved the attitude towards HCV treatment. However, follow-up of the patients referred to treatment evaluation was beyond the scope of the study, and the number of patients starting HCV treatment was not recorded.

Data on prevalence, including genotype distribution for different high-risk populations in Norway, are scarce. This is especially true for regions outside of Oslo. In order to get a comprehensive overview of the epidemic in high-risk groups in Norway, data from other regions are also needed. In this study, we prospectively collected samples from three high-risk populations. The 40% anti-HCV prevalence among prisoners is similar to that previously reported in Finland but higher than reports from Spain, the United Kingdom, France, and Denmark.^14,24^ Fifty-two percent (32) of the prisoners reported IDU as their risk factor for HCV-infection (Table 1). The high proportion of prisoners who are HCV viraemic, in combination with greater accessibility to the healthcare system, highlights that prisoners are a high-risk group for which outreach programmes are more easily administrated and for which targeted efforts can have great impact on HCV prevalence. The use of illegal drugs is prohibited in prisons and NEPs are thus not available, but the patients can be included in OST programmes by the prison healthcare department.

Globally, the anti-HCV prevalence among Norwegian PWUD was high, although lower than in a recent study from NEP in Stockholm, Sweden.^8,25^ In line with the global genotype, distribution 1a and 3 were the most common genotypes in PWUD,^26^ with genotype 3 being the dominating genotype in PWUD and prisoners in Trondheim. The majority of the PWUD patients with a history of IDU had been injecting for >10 years, suggesting that many of the patients in this study had been infected with HCV for years. Studies have shown that more than half will become HCV infected during the first 4 years of IDU,^8^ so we would have expected more patients to have progressed beyond minimal or mild fibrosis. HIV/HCV and HBV/HCV co-infection rates were low among prisoners and PWUD.

No immigrants were anti-HCV^+^. This could be owing to Eritrea and Sudan being the most common countries of origin since the HCV prevalence in Eritrea, and Sudan has been reported to be lower than for other parts of Africa,^27^ or that PWUD among immigrants decided not to participate in the study. Thus, these results do not suggest that screening of immigrants should be de-prioritised. The recommendation is still that HCV screening of immigrants from HCV endemic countries should be a priority.^24^

Interesting, over the duration of the study, the nurses became well known by the people who frequented the study sites. Participants in the study recommended their friends to sign up for the study too and saw it as a way to easily acquire information about whether they were infected or not. The accessibility of the testing was paramount for their interest. An outreach model for treatment at the same sites might increase the number of people available for treatment.

The benefit of using rapid antibody testing in more unpredictable patient cohorts like PWUD is the possibility for immediate follow-up with treatment counselling. The risk is that sensitivity and specificity will be lower compared with standard serology.^28^ In this study, none of the samples that tested positive using OraSure were false positive. However, only positive samples were retested using standard HCV serology, so a limitation of the study is that false negativity was not assessed. Another limitation is that the study did not reach the targeted 400 individuals for testing. This was due to a lower number of individuals at the refugee centre than expected based on previous enquiries to the centre. Thus, it is not possible to generalise the results for the immigrant group.

In conclusion, HCV prevalence is high among prisoners and PWUD. With a coordinated effort by the healthcare providers, it is possible to reach high-risk populations. The possibility of receiving health information with immediate answers and test-results was important to the participants. This interest in their own health should be possible to use when planning outreach programmes for treatment of these patients.

## Supplemental Material

sj-docx-1-tai-10.1177_20499361211053929 – Supplemental material for Hepatitis C outreach project and cross-sectional epidemiology in high-risk populations in Trondheim, Norway Click here for additional data file. Supplemental material, sj-docx-1-tai-10.1177_20499361211053929 for Hepatitis C outreach project and cross-sectional epidemiology in high-risk populations in Trondheim, Norway by Raisa Hannula, Jonas Söderholm, Therese Svendsen, Maja Skaland, Svein A. Nordbø, Harald Steinum and Jan K. Damås in Therapeutic Advances in Infectious Disease
