# Supplementary material for: Hepatitis C outreach project and cross-sectional epidemiology in high-risk populations in Trondheim, Norway
Source: Ther Adv Infect Dis. 2021 Oct 28;8:20499361211053929. doi: 10.1177/20499361211053929 (PMC8558792; doi:10.1177/20499361211053929)
Supplement: sj-docx-1-tai-10.1177_20499361211053929 – Supplemental material for Hepatitis C outreach project and cross-sectional epidemiology in high-risk populations in Trondheim, Norway [file sj-docx-1-tai-10.1177_20499361211053929.docx]

Supplemental table 1 – Country of origin individuals in the survey cohorts

|  | Immigrants (n=52) | Prisoners (n=62) | PWUD (n=267) |
| --- | --- | --- | --- |
| Afghanistan | - | 1 (2) | - |
| Argentina | - | - | 1 (<1) |
| Brazil | - | - | 2 (1) |
| Chile | - | - | 1 (<1) |
| Colombia | - | 1 (2) | - |
| Denmark | - | 1 (2) | 1 (<1) |
| Egypt | 1 (2) | - | - |
| El Salvador | - | 1 (2) | - |
| Eritrea | 30 (58) | - | - |
| Germany | - | - | 1 (<1) |
| Iceland | - | 1 (2) | 1 (<1) |
| Iran | - | 2 (3) | 1 (<1) |
| Iraq | 3 (6) | 1 (2) | - |
| Lithuania | - | 2 (3) | - |
| Morocco | - | - | 1 (<1) |
| Netherlands | - | 1 (2) | - |
| Norway | - | 45 (73) | 250 (94) |
| Poland | - | - | 1 (<1) |
| Russia | - | - | 1 (<1) |
| Senegal | - | - | 1 (<1) |
| Serbia | - | 1 (2) | - |
| Sierra Leon | - | - | 1 (<1) |
| Somalia | 4 (8) | 2 (3) | - |
| Sudan | 8 (15) | - | - |
| Syrian Arab Republic | 6 (3) | - | - |
| Turkey | - | 2 (3) | - |
| USA | - | - | 1 (<1) |
| Vietnam | - | - | 1 (<1) |
| No answer | - | 1 (2) | 2 (1) |
